# The multi-modal Australian ScienceS Imaging and Visualization Environment (MASSIVE) high performance computing infrastructure: applications in neuroscience and neuroinformatics research

**DOI:** 10.3389/fninf.2014.00030

**Published:** 2014-03-27

**Authors:** Wojtek J. Goscinski, Paul McIntosh, Ulrich Felzmann, Anton Maksimenko, Christopher J. Hall, Timur Gureyev, Darren Thompson, Andrew Janke, Graham Galloway, Neil E. B. Killeen, Parnesh Raniga, Owen Kaluza, Amanda Ng, Govinda Poudel, David G. Barnes, Toan Nguyen, Paul Bonnington, Gary F. Egan

**Affiliations:** ^1^Monash eResearch Centre, Monash UniversityClayton, VIC, Australia; ^2^Australian SynchrotronClayton, VIC, Australia; ^3^CSIROClayton, VIC, Australia; ^4^Centre for Advanced Imaging, University of QueenslandSt Lucia, QLD, Australia; ^5^The University of MelbourneMelbourne, VIC, Australia; ^6^Monash Biomedical Imaging, Monash UniversityClayton, VIC, Australia; ^7^CSIRO Preventative Health Flagship, CSIRO Computational Informatics, The Australian e-Health Research CentreHerston, QLD, Australia; ^8^Life Sciences Computation Centre, VLSCIParkville, VIC, Australia

**Keywords:** neuroinformatics infrastructure, high performance computing, instrument integration, CT reconstruction, cloud computing, Huntington's disease, Quantitative susceptibility mapping, digital atlasing

## Abstract

The Multi-modal Australian ScienceS Imaging and Visualization Environment (MASSIVE) is a national imaging and visualization facility established by Monash University, the Australian Synchrotron, the Commonwealth Scientific Industrial Research Organization (CSIRO), and the Victorian Partnership for Advanced Computing (VPAC), with funding from the National Computational Infrastructure and the Victorian Government. The MASSIVE facility provides hardware, software, and expertise to drive research in the biomedical sciences, particularly advanced brain imaging research using synchrotron x-ray and infrared imaging, functional and structural magnetic resonance imaging (MRI), x-ray computer tomography (CT), electron microscopy and optical microscopy. The development of MASSIVE has been based on best practice in system integration methodologies, frameworks, and architectures. The facility has: (i) integrated multiple different neuroimaging analysis software components, (ii) enabled cross-platform and cross-modality integration of neuroinformatics tools, and (iii) brought together neuroimaging databases and analysis workflows. MASSIVE is now operational as a nationally distributed and integrated facility for neuroinfomatics and brain imaging research.

## Introduction

The “21st century microscope” will not be a single instrument; rather it will be an orchestration of specialized imaging technologies, data storage facilities, and specialized data processing engines. Moreover, scientists increasingly require access to a wide range of imaging instruments, across multiple modalities and multiple scales, to characterize a scientific sample or perform an experiment. The Multi-modal Australian ScienceS Imaging and Visualization Environment (MASSIVE—www.massive.org.au) is a high performance computing facility that is specialized for computational imaging and visualization, and has been created to underpin this new landscape.

### The massive facility

MASSIVE has been established by Monash University, the Australian Synchrotron, the Commonwealth Scientific Industrial Research Organization (CSIRO), and the Victorian Partnership for Advanced Computing (VPAC) to support next-generation imaging and instrumentation. This facility provides computer hardware, software and expertise to drive research in the biomedical science, materials research, engineering, and neuroscience communities, and it stimulates advanced imaging research that will be exploited across a range of imaging modalities, including synchrotron x-ray and infrared imaging, functional and structural magnetic resonance imaging, x-ray computer tomography (CT), electron microscopy, and optical microscopy.

The MASSIVE project has a number of objectives. First, to provide a world-class imaging and visualization facility to research groups identified by the MASSIVE stakeholders. Second, to increase the uptake of imaging and visualization services by research groups using the Australian Synchrotron and by Australian research groups more generally. Third, to increase the performance and capability of imaging and visualization systems, especially the on-line reconstruction of images generated by the Imaging and Medical Beamline (IMBL) at the Australian Synchrotron. And fourth, to increase the capabilities of research groups to use and develop imaging and visualization services.

**Figure 1:**
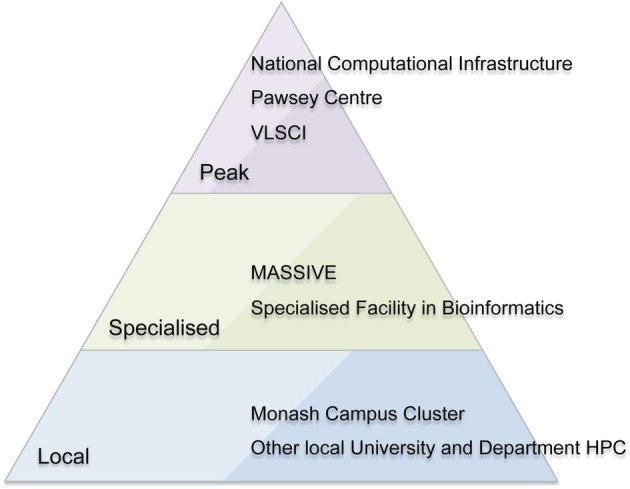
**The Australian high performance computing (HPC) environment including peak (national) facilities, specialized national facilities, and local HPC facilities**.

MASSIVE is a unique Australian facility with a focus on fast data processing, including processing data “in-experiment,” large-scale visualization, and analysis of large-cohort and longitudinal research studies. It provides this service within a national context of peak and specialized HPC facilities (Figure 1). The facility runs an instrument integration program to allow researchers to more easily process imaging data, and provides a high-performance managed interactive desktop environment providing access to common interactive analysis and visualization tools. MASSIVE offers Australian scientists access to two specialized computing facilities at Monash University and Australian Synchrotron with computer systems linked by a high-bandwidth communications link.

MASSIVE also manages a major nationally funded software infrastructure collaboration to make scientific tools, and in-particular neuroinformatics tools, available freely and cloud-ready. This collaboration, which is called the Characterization Virtual Laboratory, is composed of members of the Australian Characterization Council, the Australian Synchrotron, the Australian Nuclear Science and Technology Organization (ANSTO), the Australian Microscopy and Microanalysis Research Facility (AMMRF) and the National Imaging Facility (NIF), as well as Monash University, the University of Queensland, the Australian National University, and the University of Sydney. MASSIVE is participating in this project to support new imaging research disciplines in applying HPC, and to further develop the interactive analysis and visualization component of MASSIVE.

The total cost of MASSIVE exceeded AUD$5 million with additional contributions from the Australian Synchrotron, Monash University, CSIRO and VPAC, and is initially operational for three years until mid 2014. The MASSIVE facility is also part funded the National Computational Infrastructure (NCI) to provide imaging and visualization high performance computing facilities to the Australian scientific community. This agreement designates MASSIVE as the NCI Specialized Facility for Imaging and Visualization and allows researchers across Australia to access it based on merit allocation.

A Collaboration Agreement underpins the governance arrangements and includes a Steering Committee with an independent chair and members who are representatives of the partner organizations. The committee is guided by two Science Advisory Committees, which are the Synchrotron Science Advisory Committee and the Imaging and Visualization Advisory Committee. The facility provides an extensive program of user support and training on all aspects of high performance computing, and has an active outreach program to ensure that the MASSIVE stakeholders, Australian and international researchers, government and the broader community are aware of its benefits and achievements.

### MASSIVE and applications to neuroscience and neuroinformatics

Advanced imaging instruments, including CT and MRI scanners and electron and optical microscopes, are capable of producing data at an incredible rate. As an example, the Australian Synchrotron Imaging Beamline is able to produce data at over 500 Mbytes/s. This introduces obvious challenges for researchers to capture, process, analyze, and visualize data in a timely and effective manner. Researchers are also increasingly eager to perform data analysis “in-experiment” so that they can make appropriate decisions in real-time. MASSIVE provides real-time imaging support as follows:

- Integration of the data sources (the instruments) with the data storage and data processing engines (MASSIVE or other HPC facility) including an instrument integration support program for this purpose; and
- Provision of a common desktop environment for data processing, analysis, and visualization that is integrated with the HPC capability, and allows researchers to access their data through an environment that supports both the desktop and HPC tools they use to process their data.

This configuration results in researchers moving their data only once, automatically during data capture, with subsequent processing, analysis, and visualization performed centrally on MASSIVE. The outcome is that MASSIVE is able to support communities that have not traditionally used HPC computing.

MASSIVE currently supports over 25 Australian neuroinformatics research projects that include researchers who are:

- Undertaking large-cohort studies and longitudinal studies such as the ASprin in Reducing Events in the Elderly (ASPREE) study (Nelson et al., 2008) and the IMAGE-HD Huntington's disease study (Georgiou-Karistianis et al., 2013);
- Processing, analysing, and viewing data generated by advanced imaging equipment, including the Australian Synchrotron Imaging Beamline, new generation Computed Tomography (CT), Magnetic Resonance Imaging (MRI), and other techniques;
- Applying computer tomography techniques or volume visualization and analysis techniques;
- Applying advanced image processing, image analysis, or visualization techniques, or undertaking research in these fields; and
- Developing modeling and simulation applications, in particular applications that are suited to fast file system access or GPU hardware.

## Computing infrastructure for neuroinformatics

Scientific applications of HPC, cloud and grid computing have been thoroughly documented and computing is considered an essential scientific tool (Foster and Kesselman, 2003). A number of specialized undertakings for bioinformatics, and more specifically neuroinformatics, have been very successful and deserve particular comment.

The Biomedical Informatics Research Network (BIRN) (Grethe et al., 2005) is an infrastructure to help communities build virtual organizations, and includes support for data sharing, security, authentication and authorization, and scientific workflows. The Functional Bioinformatics Research Network (fBIRN) is a specific application of BIRN for neuroimaging, allowing researchers to calibrate and collect fMRI data across sites, and manage and analyse that data (Greve et al., 2010). Similarly, CBRAIN and GBRAIN (Frisoni et al., 2011) are an online collaborative web platform for neuroimaging allowing users to access a wide range of participating HPC resources, in Canada and across the globe.

A number of projects provide dedicated HPC access and support to neuroimaging researchers. These include the NeuGrid Redolfi (Redolfi et al., 2009) and it's successor N4U (Haitas and Glatard, 2012), and the NeuroScience Gateway (NSG) (Sivagnanam et al., 2013). All three projects provide web-based mechanisms for data management and processing and analysis on HPC systems, and specialized support for neuroimaging.

In addition there are a number of online and desktop workflow environments that are being applied to general science and specific bioinformatics and neuroinformatics purposes. These include Galaxy (Giardine et al., 2005), the LONI Pipeline (Rex et al., 2003), Kepler (Ludäscher et al., 2006), and Soma-workflow (Laguitton et al., 2011). These projects all provide mechanisms to interface with high performance computing resources. Nipype (Gorgolewski et al., 2011) is a workflow for interfacing with a range of neuroinformatics packages, allowing users to easily compare algorithms across packages. PSOM (Bellec et al., 2012) is a workflow engine for Octave and Matlab developed for neuroimaging.

The Blue Brain Project (Markram, 2006) is undertaking to simulate the brain on a HPC. The project commenced by undertaking to simulate a cellular-level model of a 2-week-old rat somatosensory neocortex based on captured microscopy data, specifically targeting the IBM Blue Gene HPC platform. This project, has now evolved into the broader Human Brain Project (HBP, 2012), which is discussed in Section Large-scale International Initiatives.

MASSIVE shares many of the fundamental goals of these projects—to provide neuroscience researchers with access to high performance computing capabilities and data management. However, our project differs in a number of ways:

- Integration of scientific instrumentation is a key feature of the project, allowing scientists to perform sophisticated processing immediately after data capture, and in some cases performing data processing as part of the experiment (Section Instrument Integration Program);
- Easy access for non HPC-experts is important to support the broad neuroscience community. Many of the projects discussed approach this problem by providing access to web portals or workflow environments. MASSIVE has decided to take the approach of providing a remote desktop (Section Massive Interactive Software Environment), which has proved effective in helping researcher transition from their personal desktop to a HPC environment. It also alleviates the need to wrap tools in a web front-end and means that a vast range of desktop tools can be supported on the systems.
- We are actively developing the MASSIVE software stack to the cloud (Section Neuroinformatics in the Cloud) which will make MASSIVE more accessible to a wider range of neuroscientists.

## Infrastructure

### Hardware

MASSIVE consists of two interconnected computers, M1, and M2 respectively, that operate at over 5 and 30 teraflops^1^ respectively, using traditional CPU processing, and accelerated to over 50 and 120 teraflops^1^, respectively, using co-processors. M1 and the first stage of M2 were made available to Australian researchers in May 2011. The computers are connected using a dedicated connection for fast file transfer and common management. A summary of the technical specifications of the two systems and the hardware configuration of the two computers, including the GPU coprocessors and the parallel file systems, are given in Table 1.

**Table 1:** **Technical specifications of the MASSIVE high performance computing system**.

**M1 AT THE AUSTRALIAN SYNCHROTRON**
42 nodes (504 CPU-cores total) in one configuration:
42 nodes with 12 cores per node running at 2.66 GHz
48 GB RAM per node (2016 GB RAM total)
2 NVIDIA M2070 GPUs with 6GB GDDR5 per node (84 GPUs total)
153 TB of fast access parallel file system
4x QDR Infiniband Interconnect
**M2 AT MONASH UNIVERSITY**
118 nodes (1720 CPU-cores total) in four configurations:
32 nodes with 12 cores per node running at 2.66 GHz
48 GB RAM per node (1536 GB RAM total)
2 × NVIDIA M2070 GPUs with 6 GB GDDR5 per node (64 GPUs total)
10 nodes with 12 cores per node (visualization/high memory configuration)
192 GB RAM per node (1920 GB RAM total)
2 × NVIDIA M2070Q GPUs with 6 GB GDDR5 per node (20 GPUs total)
56 nodes with 16 cores per node running at 2.66 GHz
64 GB RAM per node (3584 GB RAM total)
2 × NVIDIA K20 (9 nodes—18 GPUs total)
2 × Intel PHI (10 nodes—20 coprocessors total)
20 nodes with 16 cores per node running at 2.66 GHz
128 GB RAM per node (2560 GB RAM total)
2 × NVIDIA K20 (40 GPUs total)
345 TB of fast access parallel file system
4 × QDR Infiniband Interconnect
Combined the M1 and M2 have 2,224 CPU-cores.

GPUs have proved an important part of the MASSIVE environment. Key applications, including the X-TRACT (Gureyev et al., 2011) CT reconstruction software, have been parallelized to take advantage of the GPUs. This has been critical to performing fast processing of data in a near real-time fashion as discussed in Section Instrument Integration Program. Moreover, GPUs have become an important developmental technology for the research community and MASSIVE has supported a number of projects to successfully port imaging analysis code to the GPU environment. Section GPU reconstruction of quantitative magnetic susceptibility maps of the human brain describes a specific example of the application of GPUs to Quantitative Susceptibility Mapping (QSM). Importantly, the GPU capability allows MASSIVE to provide good support for interactive visualization, including through the MASSIVE Desktop (Section MASSIVE Interactive Software Environment) and through parallel rendering tools such as Paraview (Henderson et al., 2004).

Both M1 and M2 have a GPFS (Schmuck and Haskin, 2002) file system that is capable of a combined 5 GB+ per second write speed. This capability has proved essential to support both the fast capture of data from instruments, and file system intensive image processing workloads. Section Instrument Integration Program discusses the importance of the file system to support large-scale and real-time CT reconstruction image processing applications.

### Instrument integration program

**Figure 2:**
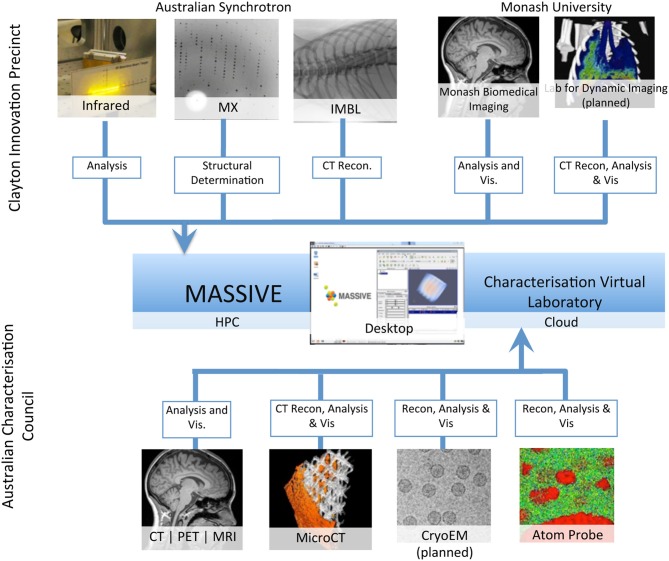
**A schematic of the integration of access to imaging instrumentation from the MASSIVE desktop and the Cloud via the Characterization virtual laboratory**.

MASSIVE has a dedicated program for the integration of imaging instruments with high performance computing capability (Figure 2, Table 2) that gives scientists the ability to use complex and computationally demanding data processing workflows within minutes of acquiring image datasets. Instruments integrated with MASSIVE that are of particular interest for neuroscience research include MRI and CT equipment at Australian National Imaging Facility locations across Australia, and for near real-time CT image reconstruction on the Imaging Beamline at the Australian Synchrotron.

**Table 2:** **The computational systems and file system access associated with the imaging instrumentation integrated with MASSIVE and the Characterization Virtual Laboratory**.

**Instrument**	**Capture method**	**Service**	**Scientific capability**
**INTEGRATED**			
Imaging and Medical Beamline	File system integration	GPU processing, parallel FS, and interactive visualization	CT reconstruction and visualization
Macromolecular Crystallography Beamline	File system mount	Compute	Structural determination
Infrared Beamline		Compute	Signal correction
X-ray Fluorescence Microprobe Beamline		Parallel FS and interactive visualization	Analysis
Small Angle and Wide Angle X-ray Scattering		Compute	Modeling
CT and MRI Imaging Instruments	DaRIS	GPU processing, parallel FS, and interactive visualization	Data capture, analysis, and visualization
Electron Microscopes	Tardis	Parallel FS, cloud computing, and interactive visualization	Data capture, analysis, and visualization
**PLANNED OR IN PROGRESS**			
Biomedical X-ray sources	File system mount	GPU processing, parallel FS, and interactive visualization	CT reconstruction and visualization
Atom Probes	Tardis	Cloud computing and interactive visualization	Analysis
Electron Microscopes	Tardis	GPU processing, parallel FS, and interactive visualization	Structural determination and visualization
Micro-CT X-ray sources	File system mount		CT reconstruction and visualization
Soft X-ray Beamline			CT reconstruction

The instrument integration program allows scientists to visualize and analyse collected data as an experiment progresses or shortly after it completes, thereby integrating processing, analysis and visualization into the experiment itself. In particular, groups that are imaging live anesthetized animals must be able to establish whether a previous scan has successfully produced the desired data before proceeding with the next step of the experiment. These experiments are typically time-critical as there is limited instrument availability once an experiment has commenced. In many cases the images captured by detectors at the Imaging Beamline are very large and necessitate the rapid movement of TB data sets for processing. These constraints dictate that significant computing power is required on demand and that the computer is tightly coupled to the instruments and readily available to the researchers.

#### Data management at monash biomedical imaging

**Figure 3:**
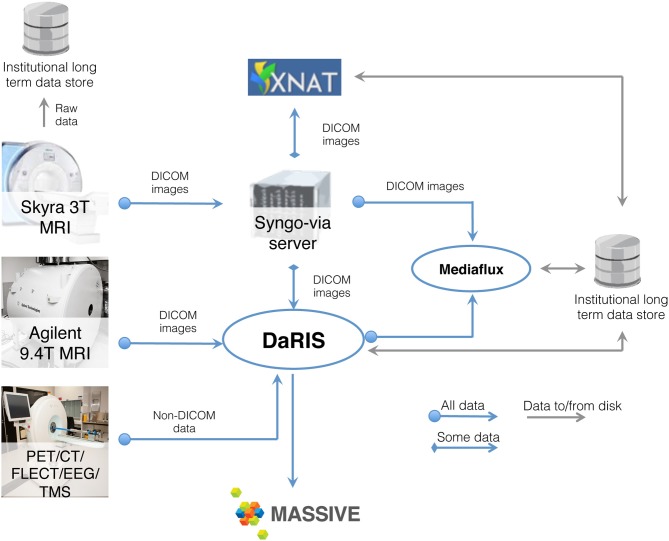
**Schematic of the neuroscience image data flow from Monash Biomedical Imaging and the computational processing performed on M2**.

Neuroimaging studies, especially multi-modal, longitudinal studies of large cohorts of subjects, generate large collections of data that need to be stored, archived, and accessed. MRI based studies can easily accumulate terabytes of data annually and require integration of HPC and informatics platforms with the imaging instrumentation. Integrated systems that combine data, meta-data, and workflows are crucial for achieving the opportunities presented by advances in imaging facilities. Monash University hosts a multi-modality research imaging data management system that manages imaging data obtained from five biomedical imaging scanners operated at Monash Biomedical Imaging (MBI) (Figure 3). In addition to Digital Imaging and Communications in Medicine (DICOM) images, raw data and non-DICOM biomedical data can be archived and distributed by the system. Research users can securely browse and download stored images and data, and upload processed data via subject-oriented informatics frameworks (Egan et al., 2012) including the Distributed and Reflective Informatics System (DaRIS) (Lohrey et al., 2009; DaRIS, 2013), and the Extensible Neuroimaging Archive Toolkit (XNAT) (Marcus et al., 2007).

DaRIS is designed to provide a tightly integrated path from instrument to repository to compute platform. With this framework, the DaRIS system at MBI manages the archiving, processing, and secure distribution of imaging data (with the ability to handle large datasets) acquired from biomedical imaging scanners and other data sources. This ensures long-term stability, usability, integrity, integration, and inter-operability of imaging data. Imaging data are annotated with meta-data according to a subject-centric data model and scientific users can find, download, and process data easily. DaRIS users can export their data directly into their MASSIVE project environment for analysis.

Recent enhancement of DaRIS (Killeen et al., 2012) provides for the management and operation of workflows (using the Nimrod and Kepler technologies) with input and output data managed by DaRIS. In this way, large subject-cohort projects can robustly process (and re-process) data with attendant enhanced data provenance. Current DaRIS enhancements are focusing on additional efficient data inter-operability capabilities so that researchers can access their managed data when and where they need it.

#### Australian synchrotron imaging beamline CT reconstruction

The MASSIVE computers have been integrated with a number of beamlines at the Australian Synchrotron, and provide a range of data processing services to visiting researchers. These include: near real-time image reconstruction at the IMBL, near-real time automated structural determination at the Macromolecular Crystallography beamline, microspectroscopy at the Infrared beamline, data analysis at the Small and Wide Angle Scattering beamlines, and image analysis at the X-ray Fluorescence Microprobe Beamline. These techniques are being applied to a range of biomedical sciences and neuroimaging applications.

**Figure 4:**
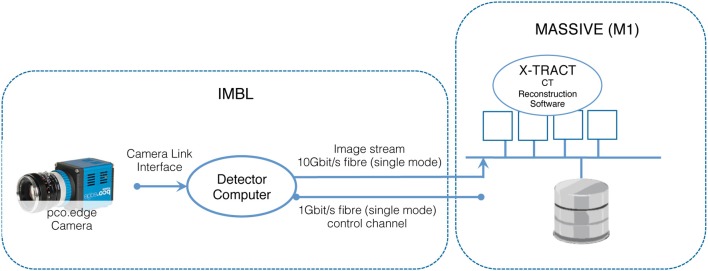
**Schematic of the architecture of the IMBL CT Reconstruction service provided on M1**.

The IMBL has a capability of near real-time high-resolution CT imaging of a range of samples, including high-resolution phase-contrast x-ray imaging of biomedical samples, animal models used in neuroscience experiments, and engineering materials. The beamline is 150 meters long, with a satellite building that includes a medical suite for clinical research as well as extensive support facilities for biomedical and clinical research programs. Two detectors based on pco-edge cameras are available for use. Typical data acquisition times are dependent upon the chosen x-ray energy and detector resolution and vary approximately between 10 and 60 min for a complete CT scan. When imaging data is acquired at the maximum detector resolution and at 50 frames per second, the data rate is 2560 × 2160 × 2 byte × 50 fps = 527.34 Mbytes/s. Figure 4 illustrates the architecture of the IMBL CT Reconstruction service.

In order to control the data collection and optimize the experimental conditions at IMBL, scientists must be able to visualize collected data in near real-time as the experiment is in progress. In particular, groups that are imaging live anesthetized animals often need to establish whether a previous scan has successfully produced the desired data before proceeding with the next step of an experiment. The experiments are typically time-critical as the window of the experiment once begun is short. The image datasets captured by detectors at the IMBL require the manipulation of data sets in the terabyte range. These experimental constraints dictate that significant computing power is tightly coupled to the experimental detectors and available on-demand.

CT data sets collected at IMBL are typically tens of GB per sample consisting of typically 1200–2400 projection images that can be acquired from a single sample in less than 1 min. The X-TRACT package on M1 is the primary software available to users for reconstruction of CT data, including phase-contrast CT as implemented at IMBL (Goscinski and Gureyev, 2011; Gureyev et al., 2011). Usage of MASSIVE for CT reconstruction via X-TRACT is offered during the synchrotron experiments and also via remote access up to 6 months after an experiment has been completed, allowing researchers to process and analyse captured data remotely. The CT reconstruction service has been in production since November 2012.

The X-TRACT customized CT image reconstruction software is parallelized for the MASSIVE GPU and parallel file system architecture. X-TRACT is an application for advanced processing of X-ray images that also provides multiple image processing tools. In particular, there is extensive functionality for X-ray CT image processing, including multiple methods for CT reconstruction and X-ray phase retrieval and simulation of phase-contrast imaging. Additionally, a large number of operations such as FFT, filtering, algebraic, geometric, and pixel value operations are provided. X-TRACT has been designed to fully leverage the processing capabilities of modern hardware such that computationally intensive operations utilize multiple processors/cores and GPU's where available to increase performance. The X-TRACT software has been adapted for use on HPC cluster infrastructure, and has been optimized for the MASSIVE systems, to enable it to process multi-TB datasets produced at synchrotron light sources that are unable to be processed on standalone desktop machines.

**Figure 5:**
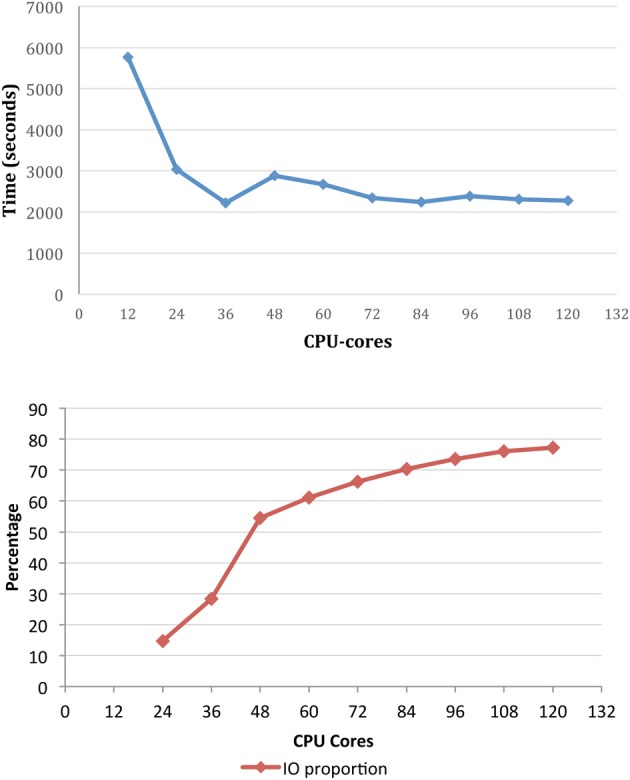
**The total reconstruction time for CT reconstruction of an 8912^3^ dataset (top) and IO time as a proportion of runtime (bottom) on M1 as a function of the number of CPU cores**.

To demonstrate the importance of the file system capability in particular, benchmarking of X-TRACT on the M1 cluster has been performed using the Gridrec CT reconstruction algorithm (Rivers and Wang, 2006) for multi-TB datasets. The total reconstruction time and IO time as a proportion of the runtime for a set of CT reconstructions is shown in Figure 5 as a function of the number of CPU cores. The input dataset consisted of 8192 pre-processed sinogram files (total ~1.5 TB), and the output was an 8192^3^ pixel dataset (total ~2 TB). The results demonstrate that IO represents a significant proportion of the overall running time—particularly beyond 36 CPU-cores. We are currently investigating the refinement of the HPC based CT processing workflow to reduce the high proportion of IO time which is currently the major performance bottleneck.

### Massive interactive software environment

**Figure 6:**
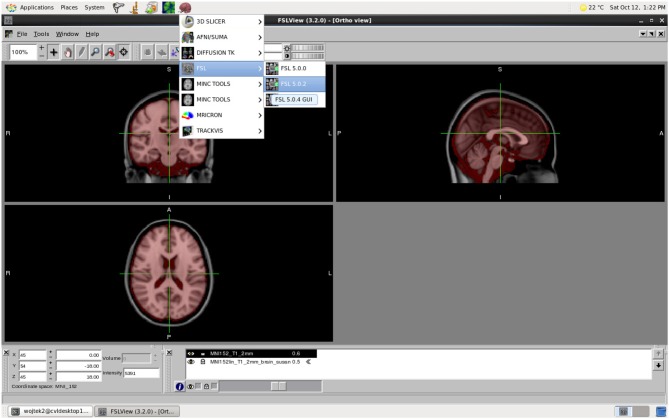
**The MASSIVE Desktop environment showing FSLView and a range of neuroinformatics tools available through the menu**.

MASSIVE provides users with highly accessible high-performance scientific desktop—an interactive environment for analysis and visualization of multi-modal and multi-scale data (Figure 6). This environment provides researchers with access to a range of existing tools and software, including commercial and open-source neuroinformatics applications. Common neuroimaging applications such as FSL (Smith et al., 2004) and SPM (Friston et al., 1994) have been integrated into the desktop to allow users to submit HPC jobs without specific HPC knowledge. The continual growth in data and study sizes increasingly necessitates the analysis and rendering of data at the location where the data is stored. Furthermore, performing analysis and visualization on a central facility greatly increases the efficiency and flexibility for researchers to access high performance hardware, including fast file systems and GPUs. Together with the MASSIVE Instrument Integration program, the desktop provides a fully integrated environment that allows researchers to view and analyze images shortly after the imaging data has been acquired.

The scientific desktop allows MASSIVE users to access a wide range of analysis tools without rewrapping or reengineering of the tools. The remote desktop has been built using CentOS running the KDE or Gnome desktop environment. For remote access, the desktop uses an open source VNC implementation, TurboVNC (http://www.virtualgl.org/), as it supports remote hardware accelerated rendering and clients on all three major platforms: Windows, Mac, and Linux. Network latency and bandwidth using the Australian academic research network (AARNET) is sufficient to support TurboVNC across the Australian imaging research community and the MASSIVE desktop is commonly accessed from every major city in Australia. The MASSIVE desktop supports a simple launcher called Strudel (short for Scientific Desktop Launcher) that automates the steps to access a desktop session. The Launcher launches an interactive visualization job on the MASSIVE system, and connects using TurboVNC using a secure SSH connection. The launcher is provided for all three major desktop platforms. It is configurable to other facilities and is being applied at other HPC facilities in Australia. It is available open source (Section Software and System Documentation).

### Neuroinformatics in the cloud

To make imaging tools more accessible to the scientific community, MASSIVE is a key participant in the Australian Characterization Virtual Laboratory (CVL) project that is funded under the National eResearch Collaboration Tools and Resources (NeCTAR) project (www.nectar.org.au). The NeCTAR CVL project is an open source project aimed at porting key scientific imaging applications to the cloud with a particular focus on neuroinformatics tools (Goscinski, 2013).

The CVL has developed a managed desktop environment, based on the MASSIVE Desktop, including the Neuroimaging Workbench to support the neuroscience imaging community. The CVL environment provides access to the MASSIVE file system and job queues and is supporting further expansion of the instrument integration program (Figure 2). The Neuroimaging Workbench has integrated workflow and database systems to allow researchers using instruments managed by the Australian National Imaging Facility (NIF) to process and manage large neuroimaging datasets. The Australian NIF is a national network of universities and biomedical research institutes that provides key biomedical imaging instruments and capabilities for the Australian research community.

Neuroinformatics tools in the cloud have great potential to accelerate research outcomes. The Neuroimaging Workbench includes a project for registration of multi-modal data brain data for the Australian Mouse Brain Mapping Consortium (Richards et al., 2011). Ultra-high resolution 15 um MRI and micro-CT images from excised tissue, can be registered with 3D reconstructions of histological stained microscopy sections. The registered datasets enable the MRI and CT images to be correlated at both the microscopic (cellular) and macroscopic (whole organ) scales. A mouse brain atlas that combines ultra-high resolution MRI and histological images has wide ranging application in neuroscience. However, image registration of 3D microscopy and MRI datasets requires immense computational power as well as a range of specialized software tools and workflows, the developed workflow is applicable to all small animal atlas building efforts.

A major objective of the CVL Neuroimaging Workbench is to increase the efficiency for the neuroimaging community to undertake complex image processing and analyses for large and longitudinal scale studies. The integration of key imaging instruments across multiple nodes of NIF is allowing neuroimaging researchers to efficiently stage data to the cloud for processing on HPC facilities. The workbench provides researchers with simple and free access to a high performance desktop environment, that contains a fully configured set of neuroimaging tools for analysis and visualization, that may obviate the need for high-end desktop workstations that are currently replicated across many neuroimaging laboratories.

### Software and system documentation

User system documentation for MASSIVE and the infrastructure developed under the Characterization Virtual Laboratory is available publically (www.massive.org.au). In addition, system documentation is available on request. Software developed under the Characterization Virtual Laboratory to support remote desktops and the neuroimaging workbench is available open source as they enter beta release (www.massive.org.au/cvl).

## Applications in neuroscience imaging

### Application to human brain imaging in huntington's disease

The IMAGE-HD study is an intensive multi-modal MRI longitudinal study in Huntington's disease (Georgiou-Karistianis et al., 2013). The IMAGE-HD study is investigating the relationships between brain structure, microstructure and brain function with clinical, cognitive and motor deficits in both pre-manifest and symptomatic individuals with Huntington's disease. Structural, functional, diffusion tensor, and susceptibility weighted MRI images have been acquired at three time points in over 100 volunteers at study entry, and after 18 and 30 months. This data is managed in the DaRIS environment. Multi-modal imaging was used to identify sensitive biomarkers of disease progression for recommendation in future clinical trials. The multi-modal imaging results have demonstrated evidence of differential rates of change in both Huntington's disease groups across a range of imaging measures with changes detected up to 15 years before the onset of symptoms (Domínguez et al., 2013; Gray et al., 2013).

The MASSIVE desktop has been used to undertake the computational imaging analyses of the structural, diffusion, and functional MRI data acquired in the IMAGE-HD study. Longitudinal diffusion tensor imaging datasets have been analyzed using deterministic (trackvis.org) and probabilistic (Behrens et al., 2007) tractography tools that have been recoded for the MASSIVE GPU and made available via the desktop. Network level brain dysfunction in axonal fiber-connectivity in HD has been analyzed using MASSIVE (Poudel et al., 2013), as well as resting-state fMRI data analyses using graph theoretical methods (Zalesky et al., 2010). The desktop is used to run semi-automated analysis pipelines for tracking longitudinal changes in structural connectivity, diffusivity in white matter, and functional connectivity in HD. The desktop is also being to used to develop combined analyses of fMRI and DTI datasets in order to understand the relationships between brain functional and microstructural deficits in Huntington's disease.

### GPU reconstruction of quantitative magnetic susceptibility maps of the human brain

Quantitative Susceptibility Mapping (QSM) (Duyn et al., 2007; Liu et al., 2009) is a technique used in MRI to measure the magnetic susceptibility of tissue, which in turn relates to the paramagnetic content of the tissue. Diffusion guided QSM (dQSM) (Ng, 2013) is a new technique that uses diffusion MRI data to improve the modeling of magnetic susceptibility at each position in the image, but it is a computationally challenging problem, requiring the inversion of a multi-terabyte matrix. Diffusion guided QSM treats the magnetic susceptibility effect of each image voxel as isotropic (Liu et al., 2011) or axial (Lee et al., 2012) depending on the fractional anisotropy (FA) in corresponding diffusion-weighted images. The computation of the matrix formulation of the problem using the Landweber iteration (LI) method is prohibitively expensive on central processing unit (CPU) cores. Acceleration of the algorithm by utilizing graphics processing unit (GPU) cores is necessary to achieve image computation times practical for research use today, and for clinical application in the near future. The dQSM problem is suited to the GPU for the reason that the elements of the matrix in the Landweber iteration formulation can be computed on-demand; without this ability the problem would be intractable on GPUs. By computing the elements of the matrix on-the-fly using the MASSIVE GPU architecture the time for computation of QSM images has been reduced by a factor of 15.

Several attributes of the Landweber iteration method applied to the dQSM problem make it particularly suitable to the GPU architecture. Computing the solution requires iteratively multiplying very large matrices, which are computed on-the-fly from smaller input buffers, with vectors of voxel input data and adding the result to the previous values. Each iteration is an Order (N^∧^2) problem with a high computational load of calculating the matrix elements that extensively uses multiply-then-add that allows fused multiply-add instructions. The conveniently contiguous access to most of the read/write data vectors by parallel computational threads enables better cache performance and reduced global memory read/write overheads. By computing the elements of the matrix on-the-fly and optimizing to best use the MASSIVE GPU architecture, the time for computation of QSM images has been reduced by a factor of 15.

The reference CPU solution uses an MPI parallel processing paradigm that already provides a domain decomposition. This decomposition was applied to the GPU implementation to split separate sections of the problem over a number of GPUs in an additional layer of parallelism. The MASSIVE architecture provides two NVIDIA Tesla M2070 or K20 GPUs per compute node along with 12 CPU cores. The fast interconnect between nodes enabled excellent scaling on the multiple GPU code with minimal communication overhead even when computed on up to 32 GPUs over 16 nodes. Current work involves a more intelligent load balancing of the work across multiple GPUs and potentially separating the problem into white-matter voxels (which require the LI technique and therefore the huge level of compute power the GPU provides), and other voxels which can be computed using a fast Fourier transform based technique. This would permit utilization of the CPU cores that sit idle while the GPU computation is performed.

The dQSM method implemented on the MASSIVE GPU architecture demonstrates greater accuracy in susceptibility estimation results compared to methods based solely on a spherical diffusion mode. The major disadvantage is the very long computation time, which makes the method challenging for routine research and clinical applications. Algorithmic improvements and the growth in compute capability of GPUs together with the further speed-up of the GPU implementation being undertaken, is expected to enable clinically-relevant post-processing times (less than 30 min). Using multi-component models of tissue structures to estimate susceptibility effects will provide more accurate results with further improvements in implementation of the dQSM algorithm.

### Digital atlasing of the mouse brain

The mouse is a vital model to elucidate the pathogenesis of human neurological diseases at a cellular and molecular level. The importance of the murine model in neuroscience research is demonstrated by the multitude and diversity of projects including the Allen Brain Atlas (brain-map.org), Waxholm Space (waxholm.incf.org) developed under the auspices of the International Neuroinformatics Coordinating Facility, the Mouse Brain Library (MBL) (mbl.org) and the Mouse Brain Architecture Project (MBAP) (brainarchitecture.org). Many research groups use non-invasive MRI to structurally map the murine brain in control and disease model cohorts. Until recently, the construction of mouse brain atlases has been relatively restricted due to the variety of sample preparation protocols and image sequences used, and the limited number of segmented brain regions.

The Australian Mouse Brain Mapping Consortium (AMBMC) has recently developed an ultrahigh resolution and highly detailed MRI-based mouse brain atlas (Richards et al., 2011; Ullmann et al., 2012). The AMBMC atlas has initially concentrated on five primary brain regions, the hippocampus, cortex, cerebellum, thalamus, and basal ganglia and has recently published a segmentation guide and probabilistic atlas for over 200 structures. MRI data from 18 C57BL/6J mice was acquired at 30 μm^3^ resolution, averaged to create a single image at a resolution of 15 μm^3^, and placed in the stereotaxic Waxholm space. The components of the brain were delineated, on the bases of differences in signal intensity and/or their location in reference to landmark structures. A digital atlas containing over 200 structures with mean region volumes, T2^*^-weighted signal intensities and probability maps for each of structure was generated for use as a detailed template for cross modality applications (see www.imaging.org.au/AMBMC).

These components have been integrated and made available through the Neuroimaging Workbench (Janke, 2013).

## Discussion and future

There are a number of major trends that will influence MASSIVE, both under its current project plan and in the future. This includes technological trends, capabilities such as visualization, and major international initiatives.

### Massive challenges

Our experience developing and managing the MASSIVE systems has highlighted a number of noteworthy challenges.

The MASSIVE systems cannot be managed in the same way as a more traditional HPC facility where computer utilization is a key measure of success. Because we commonly provide access to compute in a near-realtime or interactive manner, we must keep a proportion of the systems available and waiting for instrument processing or desktop sessions. We aim for CPU-core utilization of around 70%, as opposed to more traditional systems that are able to achieve between 90 and 100% utilization. We are experimenting with strategies such as dynamic provisioning of nodes and short running jobs to fill idle time.

Interactive desktop sessions on our facility run on a dedicated node. Thus, users have access to two CPU processors running between 8 and 12 cores, and up to 192 GB of memory. We do not allow multiple users onto a single desktop node, because a user can inadvertently affect other users. For example, by launching a multi-core application. However, a significant proportion of desktop users do not require access to the full technical capabilities. For example, a user that is using an image viewer to examine a large dataset might only require one CPU-core. The result is wasted computing resources. Our long-term plan to solve this problem is to host desktop sessions in virtual machines that will be provisioned at specific sizes and capabilities. Using virtual machines allows us to completely isolate multiple users of a single desktop and ensure a good user experience. In our early experience with provisioning on the cloud (Section Neuroinformatics in the Cloud) the overhead imposed by a virtual machine is acceptable, but fast access to file systems needs to be carefully considered.

Our most significant challenge is not technical but relates to user support. In a traditional HPC environment users will be accustomed to submitting jobs to a queue and checking back for their results. In an interactive environment, small changes to performance and accessibility have a strong effect on user experience. Moreover, users require fast response to problems—particularly considering issues with the computing system can have a major effect a physical experiment. Our solution to this problem has been to ensure that have adequate expert staff who are able to quickly triage and prioritize problems.

### Trends in scientific computing

A major trend in HPC has been the application of GPU technology, developed primarily to support the gaming market, to enable fast parallel processing. This has continued to be driven by the development of new architectures, such as the Intel Phi.

Likewise, the trend toward centralized cloud hosting, and the competition between major cloud vendors has created a landscape where hosting applications in the cloud is a very economical solution, whilst still providing a high degree of control to customize a solution to a particular science question. Early cloud hardware offerings lacked specialized hardware, such as GPUs or high performance interconnects. However, cloud computing providers are increasingly providing these capabilities, including Amazon (Ekanayake and Fox, 2010) (Amazon, 2013). In addition, the development of open source cloud computing middleware, such as OpenStack (OpenStack, 2013), allows a broader range of providers to offer cloud solutions and increases the availability of specialized services—such as parallel hardware or scientific applications. In particular, through the NeCTAR project, a number of major Australian Universities are developing an OpenStack federated multi-node cloud for the research community (NeCTAR, 2013). The CVL project is hosted on this environment allowing it access to GPUs and, in the future, a low latency and high bandwidth connection to MASSIVE. The Neuroimaging Tools and Resources Clearinghouse (NITRC) (Buccigrossi et al., 2007) Computational Environment (NITRC, 2013), is an analogous project that, like the CVL, provides a cloud platform pre-configured for neuroinformatics. This allows any neuroscientist to easily access the latest tools running on the Amazon cloud for between $0.02 and $3.10 per hour depending on the hardware configuration.

These trends in computing are creating a landscape where cloud hosting of scientific applications—including interactive desktop applications—will become a feasible, economical, and powerful solution. MASSIVE is supporting this trend by porting neuroimaging applications to the cloud through the CVL project, and integrating key Australian instruments, including the IMBL and imaging equipment through the NIF.

### Visualization for neuroinformatics

Understanding and visualizing information is a hurdle for researchers who generally work with 2D screens and rarely use 3D displays. Advances in research imaging technology has dramatically increased the volume and complexity of research data that is routinely collected. New virtual reality technologies now provide the possibility of panoramic 320° visual displays that match human visual acuity, and provide visualization opportunities for exploring, and understanding the complexity of neuroscience data, in particular human brain imaging data. The next generation of neuroscience discoveries underpinned by virtual reality technologies and advanced computational approaches have the potential to initiate a new discipline of visualization led scientific discovery in neuroscience. MASSIVE is collaborating with a unique Australian immersive visualization facility, the Monash University CAVE2 facility (CAVE2, 2013), to allow researchers to visualize MASSIVE 2D and 3D data in an immersive environment. The direct integration of the MASSIVE Desktop with the CAVE2 display facility, including support for 3D display from applications the MASSIVE users are already familiar with, is a key objective for the initial operating period of the CAVE2.

Scientists are increasingly applying a systems approach to understanding the human brain—coupling multiscale models to develop an understanding of how models work together, how effects propagate through systems and how high-level outcomes are constructed from fundamental physics and chemistry. There is a desire to provide mechanisms for interacting with and steering of simulations to understand emergent properties. In particular, the Human Brain Project (HBP, 2012; Markram, 2012) will develop mechanisms to gain visual feedback, steer simulations, and interrogate simulated models as if they were a real biological sample. New visualization tools for easily interacting with computational models, large-scale simulations, and big data are important to ensure HPC is easily accessible to the neuroscience community.

### Large-scale international initiatives

Several large-scale international brain research initiatives are now underway in both the US and Europe to accelerate our understanding of the brain and its diseases and disorders. The Human Brain Project (HBP) has been funded with the aim to take advantage of the convergence between ICT and biology to model the brain in a single multi-level system. The HBP will use supercomputers to build and simulate brain models with unprecedented levels of biological detail, and use data from new sequencing and imaging technologies, cloud technology, and neuroinformatics. The neuroinformatics community is already working closely with the large-scale initiatives to ensure collaboration on computational neuroscience and neuroinformatics standards and infrastructure.

The International Neuroinformatics Coordinating Facility (INCF) is an international organization established to coordinate international neuroinformatics infrastructure, and currently has 17 member countries across North America, Europe, Australia, and Asia. With its international network of national nodes, INCF is well positioned to connect scientists from its member countries with international large-scale brain initiatives to strengthen global collaboration and accelerate discovery in neuroscience. The INCF will play an increasingly important role in establishing and operating scientific programs to develop standards for neuroscience data sharing, analysis, modeling, and simulation. The global computational and informatics infrastructure will enable the integration of neuroscience data and knowledge worldwide, and catalyze insights into brain function in health and disease. MASSIVE participation in the Victorian node of the INCF provides an Australian centralized hardware and software facility and a national focal point for imaging and neuroinformatics expertise.

The HPB and the US-led BRAIN Initiative sit alongside a number of other major grand-challenge scientific endeavors, including mapping the human genome or understanding the fabric of matter and the universe using the CERN Large Hadron Collider or the Square Kilometer Array. These endeavors each produce immense volumes of data and are totally reliant on large-scale data processing to uncover new knowledge. Likewise, neuroscience is increasingly a data and simulation driven science and facilities such as MASSIVE are essential to develop new understandings of the brain.

## Conclusion

Neuroscience and neuroinformatics is an area of priority for the governments of most research intensive countries. Computational HPC approaches are central to neuroscience and to emerging neuroscience technologies including robotics, intelligent systems, and medical bionics. HPC facilities are essential for any future economy based on knowledge intensive industries. MASSIVE provides an Australian centralized hardware and software facility and a focal point for imaging and neuroinformatics expertise. The development of MASSIVE has been based on best practice in system integration methodologies, frameworks, and architectures. MASSIVE is now driving research in advanced brain imaging MRI, x-ray CT, optical microscopy and increasingly synchrotron x-ray and infrared imaging.

### Conflict of interest statement

The authors declare that the research was conducted in the absence of any commercial or financial relationships that could be construed as a potential conflict of interest.

## Footnotes

^1^Theoretical performance of the systems.
